# Bipolar hip hemiarthroplasty in a patient with an above knee amputation: a case report

**DOI:** 10.1186/1749-799X-4-30

**Published:** 2009-07-31

**Authors:** Leonid Kandel, Miguel Hernandez, Ori Safran, Isabella Schwartz, Meir Liebergall, Yoav Mattan

**Affiliations:** 1 Department of Orthopedic Surgery, Hadassah-Hebrew University Medical Center, Jerusalem, Israel; 2 Department of Rehabilitation and Physical Medicine, Hadassah-Hebrew University Medical Center, Jerusalem, Israel

## Abstract

The treatment of an above knee amputee who has sustained a fracture of femoral neck is a challenge for both the orthopaedic surgeon and the rehabilitation team. We present a case of such a patient and discuss different difficulties in his treatment.

## Background

Hip hemiarthroplasty is a common procedure for the treatment of subcapital femoral fractures. Good postoperative results of hip hemiarthroplasties in patients with below knee amputations have been reported in the past, with a return of the patients to their preoperative level of daily life activity [1,2]. But, to our knowledge, the use of hip hemiarthroplasty in patients with above knee amputations has not been reported in the literature.

We present a unique case of a bipolar hip hemiarthroplasty for a subcapital femoral fracture in a patient with an above knee amputation of the same extremity. The patient was informed that the case will be submitted for publication.

A 68 year old male patient was admitted to our department suffering from severe pain in his right hip joint caused by an old subcapital fracture of the femur. The patient's right leg had been amputated above the knee after a gun shot wound 58 years ago. Following the amputation he ambulated well using a fitted prosthesis – a quadrilateral socket with a swing control knee and a multiaxis foot. Six months prior to his admission, the patient fell and sustained a subcapital fracture of the right femoral neck.

**Figure 1:**
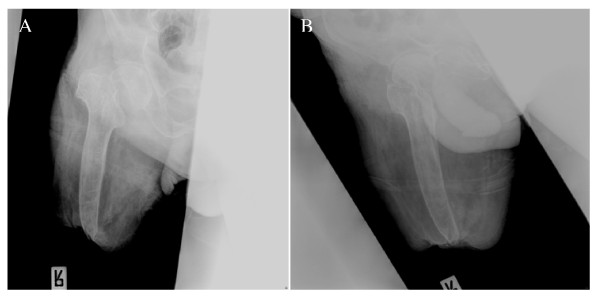
**An anteroposterior (a) and lateral (b) xrays of the fracture**.

The patient was treated in the orthopedic outpatient clinic in another country, where he received nonoperative treatment with analgesia and physiotherapy. The pain in the hip joint did not improve, and he was unable to walk. Due to the increased pain and the deterioration of the patient's daily life activity, the patient was admitted for hip hemiarthroplasty (Figure 1).

**Figure 2:**
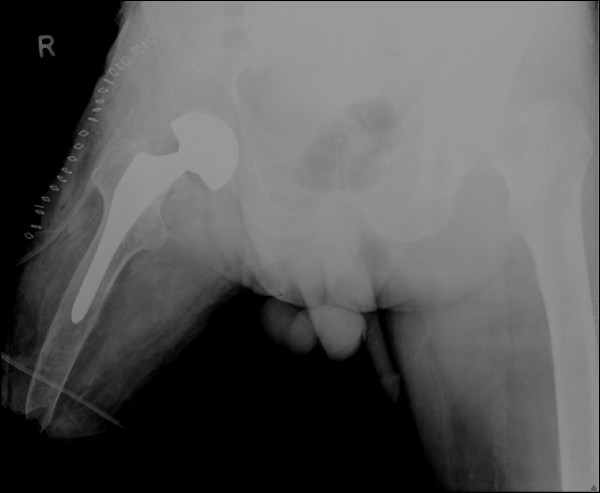
**Postoperative xray with an uncemented bipolar hemiarthroplasty**.

Under epidural anesthesia, the patient underwent bipolar hemiarthroplasty of the right hip joint (Figure 2). Because of the short lever arm of the affected femur, a bone holder was used in the subtrochanteric area to posteriorly dislocate the joint and to internally manipulate the femur during the procedure

**Figure 3:**
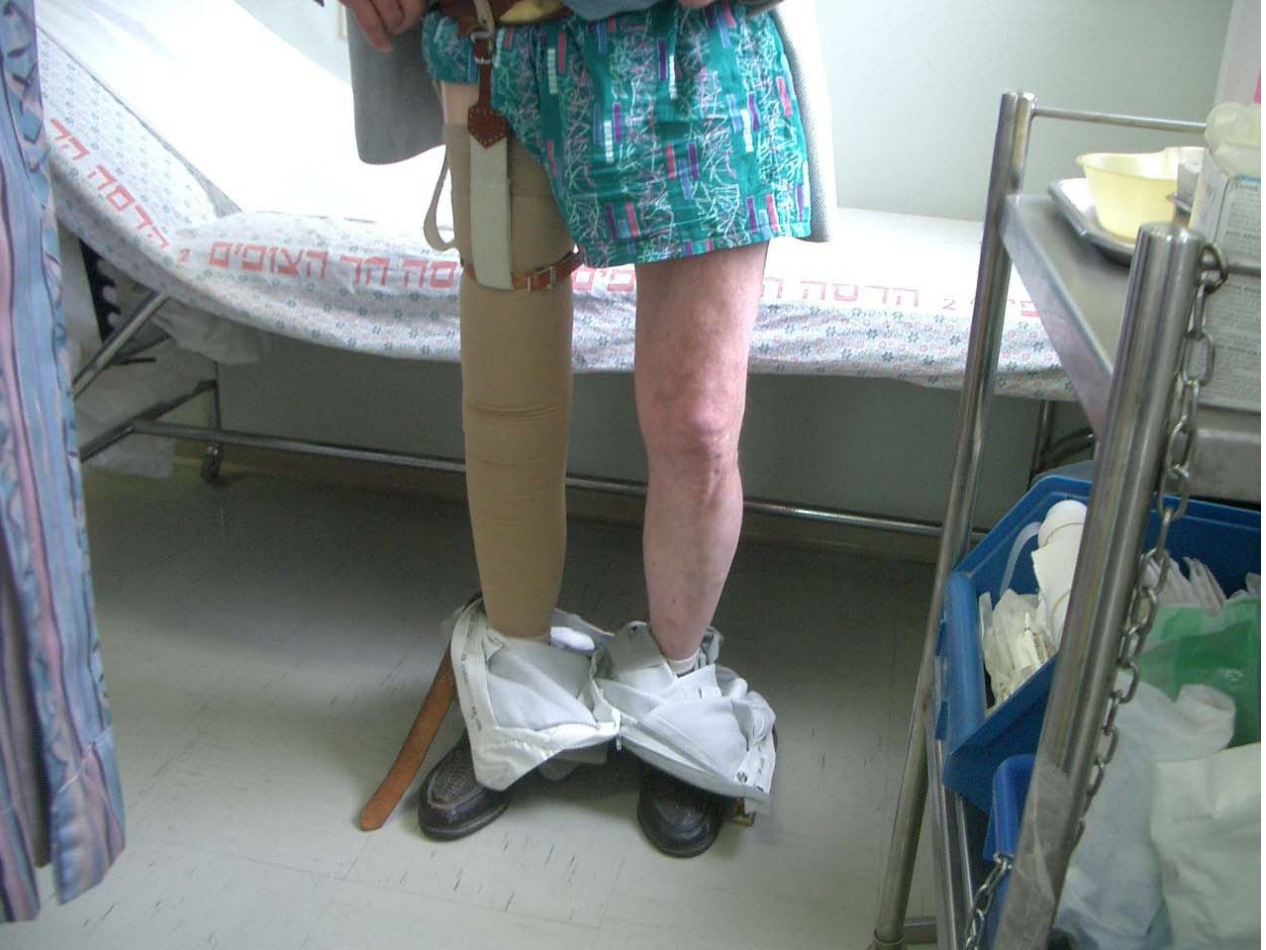
**A clinical picture with the fitted prosthesis at five years follow up**.

**Figure 4:**
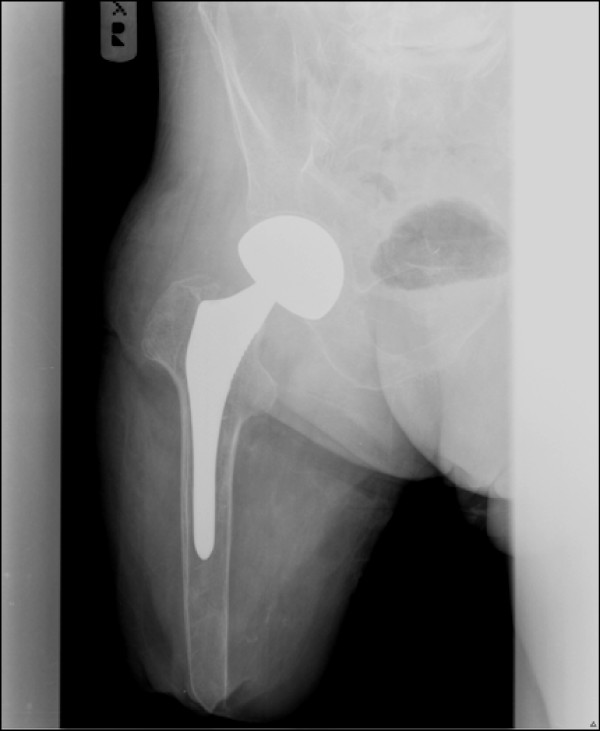
An xray five years after the hemiarthroplasty.

The postoperative course was uneventful and the patient was discharged six days later. Six weeks following surgery, the patient began partial weight bearing using the above knee prosthesis. Eight weeks after the procedure, the patient was able to put full weight bearing on the right leg with the prosthesis. During the first 3 months the use of prosthesis was uncomfortable due to significant swelling of the stump, which subsided gradually with the use of an elastic bandage. The prognosis for ambulation was good as the patient was not debilitated by other health problems and was highly motivated. A new design of socket was prescribed, a counted adducted trochanteric-controlled alignment method (CAT-CAM). This was an attempt to lock the ischial tuberosity in the socket to prevent lateral shifting and for hip joint stabilization[3]. The patient underwent six weeks course of physical therapy for prosthesis fitting and alignment. Five years after the procedure the patient ambulates well using prosthesis (a CAT-CAM socket, a swing control knee, and a multiaxis foot), with a normal gait and no pain (Figure 3). On examination he has a full range of motion, without any pain. No leg length difference was noted. The x-ray shows a normal bipolar hemiarthroplasty (Figure 4).

## Discussion

We presented an ambulating above knee amputee, who had suffered a subcapital femoral fracture. There is no clear epidemiologic data about prevalence of hip fractures in this population. Denton and McClelland[4] stated that incidence of femur and hip fractures in both above and below knee amputees. There is significant bone mass reduction of the femoral neck in amputees[5], but on the other side, the forces of the fall are lower due to decreased lever arm of the femur.

We assume that the primary treating orthopaedic surgeons expected the fracture to unite, because of the short lever arm, and the patient was treated nonoperatively. Two studies[4,6] have shown that this an appropriate strategy for most fractures after an amputation, except displaced intertrochanteric and cervical fractures that require surgical fixation. In our case, the fracture did not unite, necessitating surgical intervention.

We believe that an ambulating patient with displaced subcapital fracture will benefit more from hemiarthroplasty than from reduction and fixation of the fracture. The two major problems with this surgery are severe osteoporosis and the length of the proximal femur. The encouraging results obtained on this case were due to the technique of the surgery, especially the emphasis on the difficulties of handling the proximal femur during both the hip dislocation and the prosthesis insertion. Using a bone holder for gently holding the femur in the subtrochanteric area made the dislocation and internal rotation of the femur possible, allowing exposure of the joint and preparing the proximal femur and, if needed, the acetabulum. One should be extremely careful in using this instrument on an osteopenic femur.

Another important factor to be taken into consideration is the prevention of the swelling of the extremity after the procedure, in order to assure a prompt and complication-free return to ambulation with the prosthesis. The surgeon should protect the operating scar from the pressure of the prosthesis.

With these results, we conclude that an ambulating patient with an above knee amputation and a subcapital fracture should be operated on after appropriate planning and preparation with satisfactory results. A patient can return to preoperative level of ambulation and activity after rehabilitation.

## Consent

'Written informed consent was obtained from the patient for publication of this case report and accompanying images. A copy of the written consent is available for review by the Editor-in-Chief of this journal.'

## Competing interests

The authors declare that they have no competing interests.

## Authors' contributions

LK – conceived the idea and co-wrote the paper. MH – co-wrote the paper. OS – analyzed the notes and contributed to the discussion. IS – was responsible for the rehabilitation of the patient and wrote the rehabilitation part of the discussion. ML – performed the surgery and contributed to the discussion. YM – performed the surgery and contributed to the discussion.
